# Quantification of Heavy Metals and Pesticide Residues in Widely Consumed Nigerian Food Crops Using Atomic Absorption Spectroscopy (AAS) and Gas Chromatography (GC)

**DOI:** 10.3390/toxins13120870

**Published:** 2021-12-06

**Authors:** Kingsley O. Omeje, Benjamin O. Ezema, Finbarr Okonkwo, Nnenna C. Onyishi, Juliet Ozioko, Waheed A. Rasaq, Giacomo Sardo, Charles Odilichukwu R. Okpala

**Affiliations:** 1 Department of Biochemistry, University of Nigeria, Nsukka 410001, Enugu, Nigeria; ezema.onyebuchi@unn.edu.ng (B.O.E.); ekenedilichukwueo366@gmail.com (F.O.); sophia261@gmail.com (N.C.O.); juliet.ozioko@unn.edu.ng (J.O.); 2 Department of Applied Bioeconomy, Wrocław University of Environmental and Life Sciences, ul. Chełmońskiego 37a, 51-630 Wrocław, Poland; waheed.rasaq@upwr.edu.pl; 3 Institute for Biological Resources and Marine Biotechnology (IRBIM), National Research Council—CNR, Via Luigi Vaccara 61, 91026 Mazara del Vallo, Trapani, Italy; giacomo.sardo@irbim.cnr.it; 4 Department of Functional Foods Product Development, Faculty of Biotechnology and Food Sciences, Wrocław University of Environmental and Life Sciences, ul. Chełmońskiego 37, 51-630 Wrocław, Poland

**Keywords:** chemical toxic contaminants, consumer food safety, cereals, tubers, vegetables, maximum permissible limits

## Abstract

More still needs to be learned regards the relative contamination of heavy metals and pesticide residues, particularly those found in widely consumed Nigerian food crops like cereals, vegetables, and tubers. In this current study, the heavy metals and pesticide residues detectable in widely consumed Nigerian food crops were respectively quantified using atomic absorption spectroscopy (AAS) and gas chromatography (GC). Specifically, the widely consumed Nigerian food crops included cereals (rice, millet, and maize), legume (soybean), tubers (yam and cassava), as well as leaf (fluted pumpkin, *Amaranthus* leaf, waterleaf, and scent leaf) and fruit vegetables (okro, cucumber, carrot, and watermelon). Results showed that the detected heavy metals included arsenic (As), cadmium (Cd), chromium (Cr), cobalt (Co), iron (Fe), lead (Pb), manganese (Mn), mercury (Hg), and nickel (Ni), whereas the pesticide residues included Aldrin, Carbofuran, g-chlordane, Chlorpyrifos, DichloroBiphenyl, Dichlorodiphenyldichloroethane (DDD), Dichlorodiphenyltrichloroethane (DDT), Dichlorvos, Endosulfan, Heptachlor, Hexachlorobenzene (HCB), Isopropylamine, Lindane, t-nonachlor, and Profenofos. Across the studied food crops, the concentrations of heavy metals and pesticides were varied, with different trends as they largely fell below the established maximum permissible limits, and with some exceptions. Our findings suggest there could be a somewhat gradual decline in the concentration of the heavy metals and pesticide residues of these studied food crops when compared to previously published reports specific to Nigeria. To help substantiate this observation and supplement existing information, further investigations are required into the concentration of these heavy metals and pesticide residues specific to these studied food crops at other parts of the country.

## 1. Introduction

For the most part, fruits and vegetables provide essential phytochemicals and other nutrients to help reduce chronic ailments like obesity, diabetes, and cancers [1]. Equally, cereals and tubers comprise nutrients, such as proteins, carbohydrates, minerals, and vitamins [2] and provide economic opportunity with a view to reducing rural poverty and unemployment [3]. All the above-mentioned cultivated food crops are largely susceptible to diverse pest attacks particularly at the cultivated/farm field, which negatively influence both the economic and nutritional aspects of product quality. Obviously, controlling pest infestations requires the application of pesticides, which help to enhance farm productivity and quality [4,5,6]. Pesticides, for instance, Carbofuran, Cyanazine, Chlorotoluron, Endosulfan, Heptachlor, and Lindane, are toxicologically significant given the residues they leave behind [6,7], and together with heavy metals, they find their way through the agrofood processing and supply chain, and eventually cause various human illnesses and organ dysfunction [5,8]. Therefore, pesticides applied to agricultural crops must adhere to all applicable regulations, which ensure the remaining post-harvest chemical residues are within the consumer permissible/safe limits [6,9].

Heavy metal depicts any metallic element of an atomic weight above the mass of Ca [10]. Adding to the ingestion or exposure routes of food and water intake, the accumulation of heavy metals in cultivated crops would depend on the plant species and uptake rate [11]. Besides heavy metals as a natural form of food crop adulteration [12], their pathway into the environment would not be limited to the indiscriminate disposal of household wastes, livestock manure, and unused metallic parts [13]. The bioaccumulation of heavy metals, such as copper (Cu), cadmium (Cd), zinc (Zn), and chromium (Cr), in human tissues results in various levels of toxicity [5,14] causing illnesses like cancers, hypertension, and genetic material alteration [15,16]. When the permissible legal limits are exceeded, the heavy metals like iron (Fe), mercury (Hg), manganese (Mn), lead (Pb), and arsenic (As) exert serious health toxic effects [5,14,17]. Moreover, the consumption of vegetables, legumes, and cereals, particularly those contaminated with heavy metals, pose a risk to human health [11]. The fact that heavy metals and pesticide residues find their way through the supply chain and finally into human tissues strengthens the persistence of debate on non-regulated agrochemicals as well as the evaluation of pesticides, particularly as they concern human health and wellbeing [5]. Pesticide residues exposed through ingestion could eventually assume five orders of magnitude over those of the other routes. Human activities do contribute to increase the heavy metals concentration in the environment [18]. The fight to tackle the consumption of foodstuffs contaminated with heavy metals and pesticide residues has to intensify to prevent the resultant health risks arising from their toxicity [11].

Reports abound across Africa on the increased use of such pesticides, which involve cultivated fruit vegetables, cereals (mostly maize), legumes (e.g., cowpea), as well as tuber crops (e.g., yam, cassava) [19,20,21]. Further, the spread of heavy metals and organo-pesticides has been associated with huge mineral processing, industrial activities, and high-input agriculture [22], which has been among the key reasons for researchers to persist in the investigation of this subject area. In Nigeria, and with respect to food crops, previously reported investigations into heavy metals, together with pesticides have involved rice [23], cocoa [24], legumes, and vegetables [18,25] and have equally captured topical issues, for instance, the bioaccumulation (of heavy metals and pesticides) and associated human health risks [26,27]. Importantly, internationally recognized bodies like the United States Environmental Protection Agency (US-EPA) approves and registers the use of pesticides, as well as establishes the maximum level of pesticide chemical residues, also considered similar to the Maximum Residue Limits (MRL) in other countries [9].

Despite the available extant literature about heavy metals and pesticide residues, more still needs to be learned so as to fully understand their relative contamination levels, particularly those that involve such widely consumed Nigerian food crops like cereals, legumes, tubers and vegetables. Indeed, the contamination levels of heavy metals and pesticide residues can be quantified through the help of various rapid detection analytical methods, bioassays/chromatographic approaches, and/or omic techniques [5,7,12]. To supplement existing information, therefore, this current work quantified the heavy metals and pesticide residues detectable in cereals, fruit/leafy vegetables, and tuber crops widely consumed across Southeast Nigeria using atomic absorption spectroscopy (AAS) and gas chromatography (GC), respectively. Specifically, the studied food crops included cereals (i.e., rice (*Oryza* spp.), millet and maize (*Zea mays*)), legume (i.e., soybean), tubers (i.e., yam (*Dioscorea* spp.), and cassava (*Manihot esculenta*), as well as fruit vegetables (i.e., okro, cucumber (*Cucumis sativus* L.), carrots (*Daucus carota* L.), watermelon (*Citrullus lanatus* Thunb.)), and leafy vegetables (i.e., fluted pumpkin, *Amaranthus* leaf, waterleaf, and scent leaf). To reiterate, the AAS detects chemical elements based on light absorption by free metallic ions [28], whereas the GC identifies unknown chemical constituents even when in very tiny amounts [29]. Therefore, both ASS and GC would help to establish the respective comparative levels of heavy metals and pesticide residues detectable in such cultivated food crops like cereals, legume, tubers and vegetables.

## 2. Results and Discussion

### 2.1. Quantification of Heavy Metals

Across the studied samples, a total of eight heavy metals were detected, which include cadmium (Cd), lead (Pb), cobalt (Co), manganese (Mn), mercury (Hg), Arsenic (As), chromium (Cr), and Nickel (Ni). The maximum permissible limits established by reputable international regulators, specific to the detected heavy metals in this current work are summarized in Table 1 [30,31,32,33,34,35]. Clearly, all the maximum permissible limits of these heavy metals differ from each other, with the exception of Ni and Cr.

#### 2.1.1. Concentration of Heavy Metals Detected in the Studied Cereals (Maize, Rice, and Millet), Tubers (Cassava and Yam), and Legumes (Soybean)

The concentrations of different heavy metals (Cd, Pb, Co, Mn, Hg, As, and Ni) detected in cereals (maize, rice, and millet), tubers (cassava and yam), and legumes (soybean) are given in Table 2. When comparing the values of detected heavy metals across the studied cereals, tubers, and legume samples, some statistical differences (*p* < 0.05) were found with varying ranges of Cd from 0.000 to 0.015 mg/kg, Pb from 0.000 to 0.059 mg/kg, Co from 0.000 to 0.018 mg/kg, Mn from 0.004 to 0.146 mg/kg, Hg from 0.000 to 0.079 mg/kg, As from 0.000 to 0.028 mg/kg, as well as Ni from 0.003 to 0.023 mg/kg. Additionally, all the different heavy metals (Mn, Hg, Cd, Pb, As, and Ni) concentrations detected in the studied cereals (maize, rice, and millet) fell below the established permissible limits set by the international authorities [30,31,32,33,34,35]. Clearly, some heavy metals were not detected in some of the studied samples, which include: (a)The Pb was not detected in rice, yam; (b) The Co was not detected in maize, rice, and millet; (c)The Hg was not detected in yam, cassava, and soybean; and (d) The As was not detected in maize, yam, and soybean. Despite not being detected in rice and yam, the presence of Pb found in cassava > millet > maize> soybean should still be of consumer concern. The same can be said of Hg, which despite not being detected in yam, cassava, and soybean, its presence found in maize > millet > rice should equally be of consumer concern. The presence of Pb, for instance, could be due to it being released into the environment through lead-based gasoline combustion/painting as well as lead-containing pipes [36]. Notably also, the Hg in organic form is chemically toxic, and can bioaccumulate/biomagnify within the food chain [17].

Notably, as the As concentrations in rice (~0.028 mg/kg) and millet (~0.027 mg/kg) resembled (*p* > 0.05), both values were significantly above (*p* < 0.05) those of cassava (~0.017 mg/kg), despite being absent in maize, which was contrary to a previous report [37]. Further, the Ni concentrations in cereals herein appear much lower than the data range reported by Onianwa et al. [38] (1.00–1.89 mg/kg). It is worthy to reiterate here that Ni toxicity largely manifests as skin allergies and lung cancer [39]. Table 1 also shows that the Cd concentrations, detected in maize (~0.006 mg/kg), rice (~0.011 mg/kg), and millet (~0.008 mg/kg) were much lower than the cereal range values reported by Onianwa et al. [38] (0.06–0.28 mg/kg). It is worthy to also reiterate that high Cd levels threaten human health and could damage the kidneys and liver despite its slow excretion [40]. Table 1 also shows that the Mn concentrations detected in maize (~0.009 mg/kg) and rice (~0.004 mg/kg) were much lower than the cereal range values reported by Akinyele and Shokunbi [41] (0.06–0.28 mg/kg), with the exception of the millet sample (~0.146 mg/kg) (Table 2).

Moreover, whereas previous researchers like Onianwa et al. [38] reported Ni concentration ranges in tubers (0.93–1.79 mg/kg) and legumes (3.47–7.00 mg/kg), and Akinyele and Shokunbi [41] detected Mn in yams (~4.42 mg/kg) and soybeans (~23.42 mg/kg), Orisakwe et al. [42] reported Pb (~0.33 mg/kg), Cd (~0.10 mg/kg), and Ni (~0.30 mg/kg) in cassava, as well as Pb (~0.46 mg/kg), Cd (~0.24 mg/kg), and Ni (~1.72 mg/kg) in soybeans. Importantly, all these above-mentioned reported values were far above those obtained in this current work. Despite being a trace element that serves as an enzyme cofactor, it appears the WHO limit for Mn is not yet established. Notably, when in excess of (>5 mg/dm^3^), Mn could cause neurological disorders [43]. Moreover, cassava contains Fe, an important element responsible for a variety of biological processes, for example, oxygen transport and electron transport. When Fe is excessively consumed, however, it can lead to tissue damage [44]. The prolonged consumption of foods contaminated with high levels of Pb can be deleterious to human health and would affect the cardiovascular, nervous, skeletal, muscular, and immune systems [45].

#### 2.1.2. Concentration of Heavy Metals Detected in the Studied Fruit and Leafy Vegetables

The different concentrations of heavy metals (Co, Ni, Fe, Pb, Cr, and As) detected in fruit (okro, cucumber, carrot, and watermelon) and leafy (Ugu, *Amaranthus* leaf, waterleaf and, scent leaf) vegetables are given in Table 3. Across the studied fruit and leafy vegetables, some statistical differences (*p* < 0.05) were found when comparing the detected heavy metals values with varying ranges of Co from 0.006 to 0.064 mg/kg, Ni from 0.000 to 0.070 mg/kg, Fe from 0.038 to 0.0121 mg/kg, Pb from 0.000 to 0.338 mg/kg, Cr from 0.000 to 0.103 mg/kg, as well as As from 0.000 to 0.399 mg/kg. Further, almost all the different heavy metals (Co, Ni, Fe, Pb, Cr, and As) concentrations detected in the studied fruit and leafy vegetables fell below the established permissible limits set by the international authorities [30,31,33,34,35]. This is because, for instance, Co in okro (~0.064 mg/kg) and watermelon (~0.046 mg/kg), as well as Cr in Ugu (~0.103 mg/kg) were, respectively, above and pairing with the maximum permissible limits of 0.043 mg/kg (for Co) and 0.10 mg/kg (for Cr) set by the Food Nutrition Board and FAO/WHO [31,33,34]. Across the fruit vegetables, besides Cr detected only in carrot, the Pb concentrations in carrot (Pb = ~0.338 mg/kg) were above watermelon (Pb = ~0.240 mg/kg) then followed by cucumber (Pb = ~0.176 mg/kg). Besides a natural constituent of many anthropogenic activities, Co remains a cofactor of vitamin B_12_. Additionally, human Co consumption can come from green leafy vegetables and fresh cereals [46,47,48,49].

When heavy metals pollute both the atmosphere and soil, the development and motorization of pollutants will inevitably be deposited in plants, which would eventually find their way into the food chain [43]. Additionally, the Pb concentration peaked in carrot (~0.338 mg/kg), subsequently followed by watermelon (~0.240 mg/kg), cucumber (~0.174 mg/kg), and okro (~0.148 mg/kg). Besides being a recalcitrant environmental pollutant, and given its continuous production/release, Pb toxicity can cause a lot of harm to the human body to bring about such illnesses as anemia, as well as brain/kidney damage [36]. The Fe concentration peaked in okro (~0.053 mg/kg), followed by cucumber (~0.046mg/kg), carrot (~0.039 mg/kg), and watermelon (~0.038 mg/kg). In the human body, the Fe is among the important elements responsible for a variety of biological processes (oxygen transport and electron transport). However, an excess Fe concentration can damage some very important body tissues [44]. Onianwa et al. [38] reported Cd and Ni concentrations in some fruity vegetables, such as okro (Cd = ~0.34 mg/kg; Ni = ~2.72 mg/kg), carrot (Cd = ~0.62 mg/kg; Ni = ~9.20 mg/kg), and watermelon (Cd = ~0.01 mg/kg; Ni = ~0.80 mg/kg), as well as leafy vegetables like pumpkin leaves (Cd = ~0.22 mg/kg; Ni = ~1.40 mg/kg) and waterleaf (Cd = ~0.21 mg/kg; Ni = ~1.33 mg/kg). Essentially, these Cd and Ni values reported by Onianwa and colleagues were above those found in this current work (Refer to Table 3) where, for instance, the Ni concentration peaked in carrot (~0.07 mg/kg), followed by okro (~0.049 mg/kg), cucumber (~0.018 mg/kg), and watermelon (~0.011 mg/kg). Opaluwa et al. [37] reported Co concentrations in okro as ~0.32 mg/kg, which are well above the values detected in this current work.

Furthermore, Table 3 shows the As peak detected in cucumber (~0.168 mg/kg), which was followed by carrot (~0.120 mg/kg), okro (~0.093 mg/kg), and watermelon (~0.045 mg/kg). These specific (above-mentioned) As concentration values fell below those reported in another study [50]. The As toxicity, through contaminated food/water ingestion, threatens human life with ailments such as cancer, cirrhosis, etc. [51,52]. Moreover, Table 3 shows the *Amaranthus* leaf obtained almost all the detected heavy metals, except the Cr. Largely, the source of the heavy metal Cr can emerge from textiles, tanneries, and metallurgical industrial wastes [53]. Known for its allergenicity and carcinogenicity, the heavy metal Cr can gain entry into the human body via oral, dermal, and inhalation routes [54]. Across the leafy vegetables, the *Amaranthus* leaf obtained the highest As concentration (~0.399 mg/kg) followed by waterleaf (~0.316 mg/kg). The high As concentration in the environment can be attributed to the extensive use of As-rich fertilizers [50,55]. It is important to reiterate that these studied fruit vegetables (okro, cucumber, carrot, and watermelon) and leafy vegetables (ugu, *Amaranthus* leaf, waterleaf, and scent leaf) are intensively consumed daily around various communities in Nigeria.

### 2.2. Quantification of Pesticide Residues

A total of thirteen pesticide residues were detected across the studied samples in this current work, which include Isopropylamine, Hexachlorobenzene (HCB), Endosulfan, Aldrin, Dichlorodiphenyltrichloroethane (DDT), Profenofos, t-nonachlor, Carbofuran, Dichlorvos (2,2-dichlorovinyl dimethyl phosphate-DDVP), Heptachlor, g-chlordane, Chlorpyrifos, Lindane, and DichloroBiphenyl. The maximum permissible limits, as already established by reputable international regulators for the detected pesticide residues [56,57,58,59,60,61,62,63,64,65,66,67] of this current work, are summarized in Table 4. Notably also, Table 4 shows that some pesticide residues show similar maximum permissible limits, like Isopropylamine, g-chlordane, and Dichlorvos with 0.1 mg/kg, as well as Endosulfan and Profenofos with 0.5 mg/kg.

#### 2.2.1. Concentrations of Pesticide Residues Detected in the Studied Cereals (Maize, Rice), Legumes (Soybean), and Tubers (Cassava and Yam)

The concentrations of pesticide residues detected in the studied cereals (maize, rice), legumes (soybean), and tubers (cassava and yam) are given in Table 5. Clearly, all the studied legumes (soybean), cereals (maize, rice), and tubers (cassava and yam) samples contain one or more pesticide residues. Across the studied samples, besides the obtained significant differences (*p* < 0.05), as well as the resemblances (*p* > 0.05), not all the detected pesticide residues fell below the maximum permissible limits [56,57,58,59,60,61,62,63,64,65,66,67]. In particular, those that fell below the maximum permissible limits included Isopropylamine (in soybean), HCB (in all studied samples), Endosulfan (in all studied samples), Aldrin (only in cassava), Dichlorvos (only in soybean), and DichloroBiphenyl (only in maize). Additionally, the pesticide residues detected across the studied cereals, legumes, and tubers showed varying trends. For instance, the Isopropylamine trended as follows: Maize (0.2502 ± 0.00 mg/kg) > Millet (0.2363 ± 0.00 mg/kg) > Rice (0.2171 ± 0.00 mg/kg) > Yam (0.2165 ± 0.00 mg/kg) > Cassava (0.1649 ± 0.00 mg/kg) > Soybean (0.002 ± 0.00 mg/kg); whereas the HCB trended as follows: Millet (0.0432 ± 0.00 mg/kg) > Maize (0.0257 ± 0.00 mg/kg) > Rice (0.0252 ± 0.00 mg/kg) > Yam (0.0247 ± 0.00 mg/kg) ≈ Cassava (0.0247 ± 0.00 mg/kg) > Soybean (0.0230 ± 0.00 mg/kg), which differed from the t-nonachlor that trended as follows: Millet (0.249 ± 0.00 mg/kg) > Rice (0.1659 ± 0.00 mg/kg) > Maize (0.1427 ± 0.00 mg/kg) > Yam (0.1093 ± 0.00 mg/kg) > Soybean (0.043 ± 0.00 mg/kg) > Cassava (0.0006 ± 0.00 mg/kg).

We opine that these (above-mentioned) pesticides residues trends, particularly when considering their contamination levels, might be portraying some of the studied food crops with higher concentrations as more susceptible to the bioaccumulation process compared to the others. Such a bioaccumulation of pesticide residues would likely depend on the consumption intensity of a given contaminated food crop as well as the degree of contamination of pesticides residue [7]. Adeyeye and Osibanjo [68] detected high concentrations of organochlorine residues in yam (Aldrin = ~5.0 μg/kg; Dieldrin = ~24.0 μg/kg, and pp-DDE = ~13.0 μg/kg) and cassava (Aldrin = ~6.0 μg/kg; Dieldrin = ~31.0 μg/kg, and pp-DDE = ~21.0 μg/kg), which were far above those reported in this current work. Elsewhere in Africa, low DDD pesticide concentrations have equally been reported in widely consumed food crops, for instance, maize grain (~0.120 mg/kg) reported in Rwinga, Tanzania, as well as in fruits (~0.001 mg/kg) reported in Egypt [69]. Despite this, organochlorine pesticides (OCPs), such as HCB, DDD, and DichloroBiphenyl, still remain among those considered as highly persistent in the environment [7,70]. Akoto et al. [71] investigated the health risk assessment of pesticide residues in some food crops in Ejura, Ghana. These researchers found maize, for example, to be contaminated with organochlorine, organophosphates, and pyrethroid pesticides. Overall, these researchers considered Aldrin, Dieldrin, Heptachlor, Endrin, y-chlordane, and Chlorfenvinphos in maize, together with Heptachlor and p,p-DDD in cowpea as potential systemically toxic candidates to the consumers.

#### 2.2.2. Concentrations of Pesticide Residues Detected in the Studied Leafy Vegetables

The concentrations of pesticide residues detected in leafy vegetables (*T. occidentalis*, *Amaranthus* leaf, waterleaf, and scent leaf) are given in Table 6. Clearly, all the studied leafy vegetable samples contain one or more pesticide residue. In addition, there were some significant differences (*p* < 0.05) between the detected pesticide residues. However, not all the detected pesticide residues obtained concentrations below the maximum permissible limits. In particular, those that were below the permissible limits included Aldrin, DichloroBiphenyl, and Chlorpyrifos in waterleaf; Profenofos in *T. occidentalis*, DDT in *T. occidentalis* and scent leaf, Lindane in *Amaranthus* leaf, Heptachlor in *Amaranthus* leaf and scent leaf, and t-nonachlor in *Amaranthus* leaf. However, g-chlordane in waterleaf appeared either parallel or somewhat above the permissible limit. Aside from the situation of permissible limits, the detected pesticide residues across the studied leafy vegetables exhibited some trends. For instance, DichloroBiphenyl was detected in all the samples studied, peaked in *T. occidentalis* (0.0115 ± 0.00 mg/kg), followed by scent leaf (0.0112 ± 0.00 mg/kg), *Amaranthus* leaf (0.0062 ± 0.00 mg/kg), and then waterleaf (0.0059 ± 0.00 mg/kg). Besides, some pesticide residues occurred in only one leafy vegetable, for example, Lindane (0.5036 ± 0.00 mg/kg) and t-nonachlor, (0.1468 ± 0.00 mg/kg) were detected only in *Amaranthus* leaf, whilst Chlorpyrifos (0.0000 ± 0.00 mg/kg) was detected only in waterleaf.

Previous researchers like Njoku et al. [72] detected some pesticide residues in *T. occidentalis* with different concentration ranges, for example, Aldrin (0.000–0.052 mg/kg), Carbofuran (0.000–0.113 mg/kg), Endosulfan (0.000–0.087 mg/kg), and Profenofos (0.000–0.176 mg/kg), all of which corroborated the data in this current work. Elsewhere, Akan et al. [73] demonstrated that organophosphate pesticide residues like Chlorpyrifos, Diazinon, Dichlorvos, and Fenitrothionin in different vegetables (cabbage, lettuce, onion, spinach, and tomato) can be detected in agricultural areas of Borno State, Nigeria. Importantly, the risks that pesticide residues pose to human health, as reiterated by Mazlan et al. [6], must not be taken for granted. For instance, Aldrin can cause increased d-glutaric acid metabolism. Lindane can cause allergic reactions/rash, contact dermatitis, enzyme induction, as well as skin sensitisation. Also, HCB can cause bulb formation, permanent loss of hair, photosensitivity, as well as skin atrophy.

#### 2.2.3. Concentrations of Pesticide Residues Detected in the Studied Fruit Vegetables (Okro, Cucumber, Carrot, and Watermelon)

The concentrations of pesticide residues detected in the studied fruit vegetables (okro, cucumber, carrot, and watermelon) are given in Table 7. Clearly, all the studied fruit vegetable samples contain one or more pesticide residues. In addition, there were some significant differences (*p* < 0.05) comparing the pesticide residues detected across the studied samples. However, not all the detected pesticide residues obtained concentrations below the maximum permissible limits [56,57,58,59,60,61,62,63,64,65,66,67]. In particular, the situations where the permissible limits were exceeded included Aldrin and g-chlordane in carrot, DDT in okro, Lindane in cucumber, carrot, and watermelon, Heptachlor in okro, cucumber, and watermelon, as well as t-nonachlor in cucumber and watermelon. Aside from the situation of permissible limits of the detected pesticide residues in these fruit vegetables, there were some trends that were shown. For instance, Endosulfan trended as follows: Carrot (0.0733 ± 0.00 mg/kg) > Cucumber (0.0376 ± 0.00 mg/kg) > Okro (0.0147 ± 0.00 mg/kg); whereas Lindane trended as follows: Carrot (0.5171 ± 0.000 mg/kg) > Cucumber (0.5036 ± 0.00 mg/kg) > Watermelon (0.4191 ± 0.00 mg/kg); which differed from t-nonachlor that trended as follows: Cucumber (0.1468 ± 0.001 µg/mL) > Watermelon (0.0811 ± 0.00 mg/kg) > Carrot (0.0003 ± 0.00 mg/kg).

Odewale et al. [74], through investigations on four regularly consumed fruit vegetables cultivated in Southwest Nigeria, equally detected some organochlorine pesticides like Aldrin, Endosulfan, Heptachlor, and Dieldrin. These researchers found Endosulfan as the predominant organochlorine pesticide residue in carrot (~2.532 mg/kg), cucumber (~1.729 mg/kg), and watermelon (~1.154 mg/kg), all of which are above the values reported in this current work. Rakhimol et al. [7] reiterated that the likes of Aldrin, Heptachlor, and Lindane, despite being an easy pesticide to apply on the crop farm field, remain characterized mostly as neurotoxins, which would bioaccumulate, and, at the same time, be biomagnific. Table 7 also shows that HCB and Chlorpyrifos detected in watermelon (HCB = 0.0804 ± 0.00 mg/kg; Chlorpyrifos = 0.3161 ± 0.00 mg/kg) were above those of okro (HCB = 0.0253 ± 0.00 mg/kg; Chlorpyrifos = 0.2092 ± 0.00 mg/kg). Additionally, Aldrin and Profenofos both detected in carrot (Aldrin = 0.2420 ± 0.00 mg/kg; Profenofos = 0.3304 ± 0.00 mg/kg) were above those of watermelon (Aldrin = 0.0009 ± 0.00 mg/kg; Profenofos = 0.2104 ± 0.00 mg/kg). It is worthy to mention that some of the detected pesticide residues, such as Aldrin, Heptachlor, Lindane, and Endosulfan, appear previously banned by the Nigerian government, as reported by Mazlan et al. [6].

## 3. Conclusions

The increases in detected heavy metals and pesticide residues among cultivated food crops in Nigeria (applicable to other agro-thriving nations) continue to be a very key consumer/public health issue. In this current work, we demonstrated the comparative levels of heavy metals and pesticide residues in commonly consumed cultivated food crops in Nigeria, and the combined use of AAS and GC has helped to actualise this. Clearly, detectable levels of heavy metals and pesticide residues in cereals, legume, and tubers as well as in fruit/leaf vegetables were found in this current study. In many cases herein, the concentrations of heavy metals and pesticide residues fell below, not only the established maximum permissible limits [30,31,32,33,34,35,56,57,58,59,60,61,62,63,64,65,66,67], but also, the reported data of previously published studies specific to Nigeria [6,37,38,41,42,61,68,72,74,75]. These results might suggest there could be a potential/somewhat gradual decline in heavy metals and pesticide residues concentration compared to previously published reports specific to Nigeria. To help substantiate this observation, and supplement existing information, further investigations are required into the concentration of these heavy metals and pesticide residues specific to these studied food crops at other parts of the country.

Essentially, the use of maximum permissible limits is instituted given the health hazards associated with the contamination of both heavy metals and pesticide residues, and that is why the Codex Alimentarius, together with the food standards of the Food Agriculture Organization/World Health Organization (FAO/WHO), places vital emphasis on these contaminations (arising from the heavy metals and pesticide residues) [76]. That is also why the excessive consumption of these studied food crops must be with great caution, especially in the situations where higher concentrations of such heavy metals and pesticide residues, are being and/or have been detected/perceived. Future work, therefore, should be directed to wider and long-term studies into the influence of location (like rural or urban), time period (months or years), source of purchase (farm, rural market, or supermarket), etc., on the degree of heavy metals and pesticide residues contamination of these studied (and other cultivated) food crops. Additionally, future work should seek to know how farmers/the general public perceive both the degree of heavy metals and pesticide residues contamination and the increased use of such pesticides like bactericides, fungicides, herbicides, insecticides, and rodenticide utilised in various cultivated farms of fruit vegetables, cereals (mostly maize), legumes (e.g., cowpeas), as well as tuber crops (e.g., yam, cassava).

## 4. Materials and Methods

### 4.1. Schematic Overview of the Experimental Program

**Figure 1:**
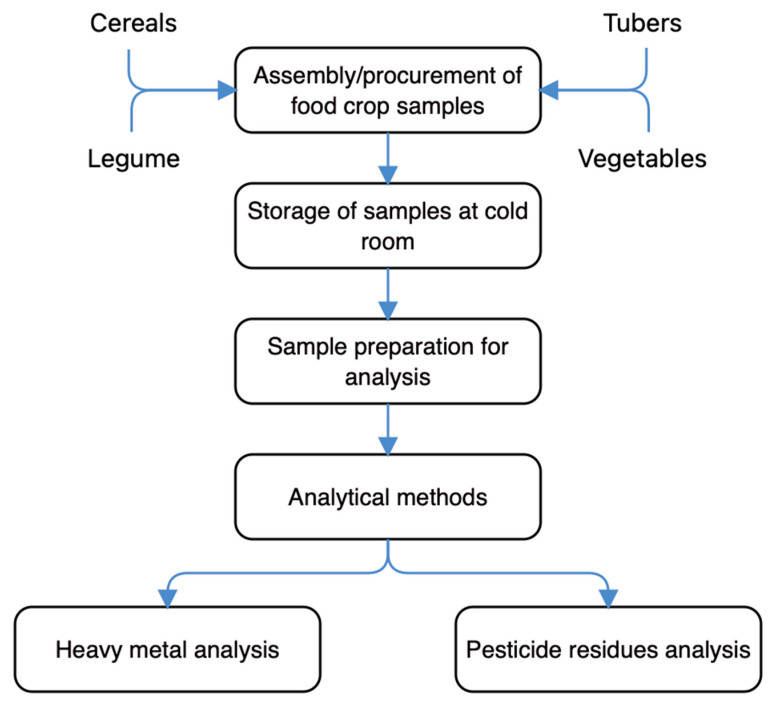
The schematic overview of the experimental program showing the key stages, from the assembly/procurement of food crop samples, the storage at cold room, sample preparation for analysis, to the quantification of detectable heavy metals and pesticide residues.

The schematic overview of the experimental program given in Figure 1 reveals the key stages of this current work, from the assembly/procurement of food crop samples, the storage at cold room, sample preparation for analysis, to the quantification of detectable heavy metals and pesticide residues. For emphasis, this current work performed an AAS and a GC quantification of heavy metals and pesticide residues that could occur in cereals, fruit, and leafy vegetables, and tuber crops commonly consumed in Southeast Nigeria. Specifically, the cereals included rice, millet, and maize; the legumes included soybean, the fruit vegetables included okro, cucumber, carrot, and watermelon; the leafy vegetables included fluted pumpkin, *Amaranthus* leaf, waterleaf, and scent leaf; and the tubers included yam and cassava. Additionally, the detected heavy metals and pesticide residues were performed on different studied samples of the same batch.

### 4.2. Procurement and Storage of Samples

Freshly harvested cereals (rice, millet, and maize), fruit vegetables (okro, cucumber, carrot, and watermelon), leafy vegetables (fluted pumpkin/ugu, *Amaranthus* leaf, waterleaf, and scent leaf), and tubers (yam and cassava) were procured from open markets situated in Nsukka and Enugu both in Enugu State (6°51′24″ N and 7°23′45″ E) situated within the Southeast geopolitical zone of Nigeria. In particular, Enugu State is believed to have an increasing population; it is realistically estimated to be at six million [77]. After purchase, all the samples were immediately subject to cold room (2–4 °C) storage until required at the laboratory of Department of Biochemistry, University of Nigeria, Nsukka.

### 4.3. Chemicals and Reagents

All the chemicals and reagents were procured from certified retailers; the chemicals and reagents included sodium sulphate, n–hexane, nitric acid (conc. HNO_3_), perchloric acid; sulphuric acid (conc. H_2_SO_4_), sodium sulphate, chloroform, benzene, as well as methanol. Importantly, the chemicals and reagents were of an analytical grade standard.

### 4.4. Sample Preparation

About 10 g of the homogenized sample was mixed with 60 g of anhydrous sodium sulphate in an agate mortar, in order to absorb the emergent moisture. Thereafter, the homogenate was placed in a 500 mL beaker, and extraction was carried out using 300 mL of n-hexane for 24 h at room temperature, following the cold extraction method adapted from Olasehinde et al. [78]. The extract was evaporated to dryness at 40 °C with the help of a rotary vacuum evaporator.

### 4.5. Analytical Methods

#### 4.5.1. Heavy Metal Analysis

The heavy metal analysis was performed with the help of a Varian AA240 Spectrophotometer (Varian Inc., Palo Alto, CA, USA) equipped with an acetylene air flame, adapting the protocol described by Quarcoo and Adotey [79] with slight modifications. The pyrolytic-coated graphite tubes of the AAS Spectrophotometer were equipped with platform instrument settings and furnace programs that helped to ascertain the peak signals. A known concentration of the sample (~2 g) was put into a digestion flask, along with 20 mL of acid mixture (which comprised 650 mL conc HNO_3_; 80 mL perchloric acid; 20 mL conc H_2_SO_4_), subsequently heated until a clear digest was obtained. The digest was diluted with distilled water to the 100 mL mark. The acid level samples as they came along were monitored by a pH meter. The disgestate was quantified, assayed for heavy metals using a Varian AA240 Spectrophotometer, and reported in mg/kg. The reference standards (Fluka Analytical, Sigma-Aldrich Chemie GmbH, Switzerland) for the detected element, blanks, and their duplicates were digested using the condition consistent with those of the samples.

#### 4.5.2. Pesticide Residues Analysis

The pesticide residues were determined with the help of a GC analysis and prepared following the AOAC [80] method with slight modifications. Ten grams (10 g) of the homogenized sample was mixed with 60 g of anhydrous sodium sulphate in an agate mortar to absorb moisture. The homogenate was transferred into a 500 mL beaker, and the extraction was carried out with 300 mL of n–hexane for 24 h. The crude extract obtained was concentrated using a rotary vacuum evaporator at 40 °C to dryness. The sample residue (1 mL) was measured into 50 mL of chloroform and transferred to a 100 mL volumetric flask and diluted. Most of the chloroform was evaporated at room temperature before adding 1 mL of the solvent mixture (20% benzene and 55% methanol). The mixture was sealed and heated at 40 °C using a water bath for 10 min. After heating, the organic sample was extracted with n–hexane and water in a proportion of 1:1. The mixture was shaken vigorously for 2 min, and n-hexane phase was transferred onto a small test tube for injection into a Buck 530 Gas Chromatograph (GC) equipped with an on-column, automatic injector, electron capture detector, and an HP 88 capillary column (100 m × 0.25 µm film thickness) (Agilent Technologies, Santa Clara, CA, USA), with injector and detector temperatures of 180 and 300 °C, respectively. Overall, the GC enabled the identification of pesticide residues, which were recorded in mg/kg, as the results emerged.

### 4.6. Statistical Analysis

The emergent heavy metals and pesticide residues data were from triplicate determinations of different samples from a given food crop batch. A one-way analysis of variance (ANOVA), using SPSS for Windows (version 16, SPSS Inc., Chicago, IL, USA) helped to establish the differences in heavy metals/pesticide residues across the studied food crop samples expressed as mean ± standard error (SE). A simple *t*-test, using GraphPad Software (https://www.graphpad.com/quickcalcs/, accessed on 19 November 2021), enabled the comparisons between the heavy metals/pesticide residues concentration data and the established/referenced maximum permissible limits. The probability level has been set at *p* < 0.05 (95% confidence level).

## Figures and Tables

**Table 1:** The maximum permissible limits established by reputable international regulators, specific to the detected heavy metals in this current work.

Heavy Metal Types	Maximum Permissible Limits	Country/Regulator	References
Cadmium (Cd)	0.3 mg/kg	Food and Agriculture Organization/World Health Organization, Joint FAO/WHO Expert Committee on Food Additives	[31,33]
Lead (Pb)	1.0 mg/kg	Food and Agriculture Organization/World Health Organization, Joint FAO/WHO Expert Committee on Food Additives	[30,31,33]
Cobalt (Co)	0.043 mg/kg	Food and Nutrition Board	[34]
Manganese (Mn)	2.0 mg/kg	-Food and Agriculture Organization/World Health Organization, Joint FAO/WHO Expert Committee on Food Additives -Codex Alimentarius Commission Food Additives and Contaminants	[30,32]
Mercury (Hg)	0.5 mg/kg	Food and Agriculture Organization/World Health Organization, Joint FAO/WHO Expert Committee on Food Additives	[32,33]
Arsenic (As)	1.4 mg/kg	Food and Agriculture Organization/World Health Organization, Joint FAO/WHO Expert Committee on Food Additives	[31,33]
Nickel (Ni)	0.10 mg/kg	-Food and Agriculture Organization/World Health Organization, Joint FAO/WHO Expert Committee on Food Additives -U.S. Environmental Protection Agency	[32,35]
Chromium (Cr)	0.10 mg/kg	Food and Agriculture Organization/World Health Organization, Joint FAO/WHO Expert Committee on Food Additives	[31,33]

**Table 2:** Concentrations of different heavy metals (Cd, Pb, Co, Mn, Hg, As, and Ni) detected in cereals (maize, rice, and millet), tubers (cassava and yam), and legumes (soybean).

Detected Heavy Metals	Maize	Rice	Millet	Yam	Cassava	Soybean	Permissible Limits with Reference Sources
Cd (mg/kg)	0.006 ^Ab^ ± 0.00	0.011 ^Bb^ ± 0.00	0.008 ^Ab^ ± 0.00	0.000	0.006 ^Ab^ ± 0.00	0.015 ^Cb^ ± 0.00	0.3 ^a^ [31,33]
Pb (mg/kg)	0.046 ^Ab^ ± 0.00	0.000	0.053 ^Bb^ ± 0.00	0.000	0.059 ^BCb^ ± 0.00	0.041 ^Ab^ ± 0.00	1.0 ^a^ [31,33]
Co (mg/kg)	0.000	0.000	0.000	0.003 ^Ab^ ± 0.00	0.005 ^Ab^ ±0.00	0.018 ^Bb^ ± 0.00	0.043 ^a^ [34]
Mn (mg/kg)	0.009 ^Ab^ ±0.00	0.004 ^Ab^ ± 0.00	0.146 ^Bb^ ± 0.00	0.066 ^Cb^ ± 0.00	0.072 ^Db^ ± 0.00	0.077 ^Db^ ± 0.00	2.0 ^a^ [30,32]
Hg (mg/kg)	0.079 ^Ab^ ± 0.00	0.068 ^Ab^ ± 0.00	0.069 ^Ab^ ± 0.00	0.000	0.000	0.000	0.5 ^a^ [32,33]
As (mg/kg)	0.000	0.028 ^Ab^ ± 0.00	0.027 ^Ab^ ± 0.00	0.000	0.017 ^Bb^ ± 0.00	0.000	1.4 ^a^ [31,33]
Ni (mg/kg)	0.009 ^Ab^ ± 0.00	0.003 ^Ab^ ± 0.00	0.014 ^Bb^ ± 0.00	0.009 ^Ab^ ± 0.00	0.023 ^Cb^ ± 0.00	0.021 ^Cb^ ± 0.00	0.10 ^a^ [32,35]

Key: Each value from triplicate determinations, presented in mean ± standard error (SE); values with different superscripts (lowercase [^a,b^]) are statistically significant (*p* < 0.05) compared to the maximum permissible limits; values with different superscripts (uppercase [^A–D^]) are statistically significant (*p* < 0.05) across samples (of same heavy metals).

**Table 3:** Concentrations of heavy metals (Co, Ni, Fe, Pb, Cr, and As) detected in fruit vegetables (*okro*, cucumber, carrot, and watermelon) and leafy vegetables (Ugu, *Amaranthus* leaf, waterleaf, and scent leaf).

Detected Heavy Metals	Okro	Cucumber	Carrot	Watermelon	Ugu	Amaranthus	Waterleaf	Scent Leaf	Permissible Limits with Reference Sources
Co (mg/kg)	0.064 ^Ac^ ± 0.00	0.027 ^Ba^ ± 0.00	0.034 ^Ba^ ± 0.00	0.046 ^Cb^ ± 0.00	0.009 ^Da^ ± 0.00	0.008 ^Da^ ± 0.00	0.006 ^Da^ ± 0.00	0.018 ^Da^ ± 0.00	0.043 ^b^ [34]
Ni (mg/kg)	0.049 ^Ab^ ± 0.00	0.018 ^Bb^ ± 0.00	0.070 ^Ab^ ± 0.00	0.011 ^Bb^ ± 0.00	0.034 ^Cb^ ± 0.00	0.049 ^Ab^ ± 0.00	0.000	0.028 ^Bb^ ± 0.00	0.1 ^a^ [32,35]
Fe (mg/kg)	0.053 ^A^ ± 0.00	0.046 ^A^ ± 0.00	0.039 ^B^ ± 0.00	0.038 ^B^ ± 0.00	0.105 ^C^ ± 0.00	0.079 ^A^ ± 0.00	0.121 ^C^ ± 0.00	0.068 ^A^ ± 0.00	N/A
Pb (mg/kg)	0.148 ^Ab^ ± 0.00	0.176 ^Ab^ ± 0.00	0.338 ^Bb^ ± 0.00	0.240 ^Cb^ ± 0.00	0.000	0.106 ^Ab^ ± 0.00	0.008 ^Db^ ± 0.00	0.000	1.0 ^a^ [30,31,33]
Cr (mg/kg)	0.000	0.000	0.093 ^Ab^ ± 0.00	0.000	0.103 ^Aa^ ± 0.00	0.000	0.029 ^Bb^ ± 0.00	0.041 ^Bb^ ± 0.00	0.10 ^a^ [31,33]
As (mg/kg)	0.120 ^Ab^ ± 0.00	0.168 ^Ab^ ± 0.00	0.045 ^Bb^ ± 0.00	0.000	0.000	0.399 ^Cb^ ± 0.00	0.316 ^Cb^ ± 0.00	0.080 ^Bb^ ± 0.00	1.4 ^a^ [31,33]

Key: Each value from triplicate determinations, presented in mean ± standard error (SE); N/A = Not available; values with different superscripts (lowercase [^a–c^]) are statistically significant (*p* < 0.05) compared to the maximum permissible limits; values with different superscripts (uppercase [^A–D^]) are statistically significant (*p* < 0.05) across samples (of same heavy metals).

**Table 4:** The maximum permissible limits, as already established by reputable international regulators, for the detected pesticide residues [56,57,58,59,60,61,62,63,64,65,66,67] of this current work.

Pesticide Residue Types	Maximum Permissible Limits	Reputable International Regulators	References
Isopropylamine	0.1 mg/kg	United States Environmental Protection Agency (EPA)	[56]
DichloroBiphenyl (also considered among ‘polychlorinated biphenyls’)	0.2 mg/kg	Commission Regulation (EC) No 1881/2006. Setting maximum levels for certain contaminants in foodstuffs”	[57]
HCB (Hexachlorobenzene)	0.5 mg/kg	United States Environmental Protection Agency (EPA)	[58]
Endosulfan	0.5–5 mg/kg	FAO Working Party of Experts and the WHO ExpertCommittee on Pesticide Residues	[59]
Aldrin	0.2 mg/kg	FAO/WHO Joint Meeting on Pesticide. Residues	[60]
Profenofos	0.5 mg/kg	International Food Standards/Codex Alimentarius FAO/WHO (a)	[61]
DDT	0.01 mg/kg	Joint FAO/WHO Meeting on Pesticide Residues (JMPR) (a)	[62]
Lindane	0.02 mg/kg	Joint FAO/WHO Meeting on Pesticide Residues (JMPR)(b)	[63]
g-chlordane	0.1 mg/kg	International Programme on Chemical Safety (IPCS)	[64]
Dichlorvos (DDVP)	0.1 mg/kg	International Food Standards/Codex Alimentarius FAO/WHO (b)	[65]
Heptachlor	0.01 mg/kg	International Food Standards/Codex Alimentarius FAO/WHO (c)	[66]
t-nonachlor	0.04 mg/kg	Canadian Health Measures Survey	[67]
Chlorpyrifos	1.0 mg/kg	International Food Standards/Codex Alimentarius FAO/WHO (b)	[65]

**Table 5:** Concentrations of pesticide residues detected in the studied cereals (maize, rice), legumes (soybean), and tubers (cassava and yam).

Detected Pesticides	Maize	Rice	Yam	Cassava	Soybean	Millet	Maximum Permissible Limit and Reference Sources
Isopropylamine (mg/kg)	0.2502 ^Ab^ ± 0.00	0.2171 ^Ab^ ± 0.00	0.2165 ^Ab^ ± 0.00	0.1649 ^Ab^ ± 0.00	0.0020 ^Bc^ ± 0.00	0.2363 ^Ab^ ± 0.00	0.1 ^a^ [56]
HCB (mg/kg)	0.0257 ^Ab^ ± 0.00	0.0252 ^Ab^ ± 0.00	0.0247 ^Ab^ ± 0.00	0.0247 ^Ab^ ± 0.00	0.0230 ^Ab^ ± 0.00	0.0432 ^Bc^ ± 0.00	0.5 ^a^ [58]
Endosulfan (mg/kg)	0.0795 ^Bb^ ± 0.00	0.0796 ^Bb^ ± 0.00	0.0340 ^Ac^ ± 0.00	0.090 ^Cb^ ± 0.00	0.0000	0.0000	0.5 ^a^ [59]
Aldrin (mg/kg)	0.0239 ^Aa^ ± 0.00	0.0238 ^Aa^ ± 0.00	0.0000	0.0937 ^Bb^ ± 0.00	0.0000	0.0000	0.2 ^c^ [60]
DDT (mg/kg)	0.1124 ^Aa^ ± 0.00	0.1168 ^Aa^ ± 0.00	0.0000	0.2590 ^Bb^ ± 0.00	0.0000	0.0000	0.01 ^c^ [62]
t-nonachlor (mg/kg)	0.1427 ^Ab^ ± 0.00	0.1659 ^Ab^ ± 0.00	0.1093 ^Ac^ ± 0.00	0.0006 ^Cf^ ± 0.00	0.043 ^Bd^ ± 0.00	0.2490 ^Aa^ ± 0.00	0.04 ^e^ [67]
Carbofuran (mg/kg)	0.0000	0.2758 ^A^ ± 0.00	0.1668 ^B^ ± 0.00	0.0343 ^B^ ± 0.00	0.1530 ^A^ ± 0.00	0.0000	N/A
Dichlorvos (DDVP) (mg/kg)	0.1955 ^Aa^ ± 0.00	0.2143 ^Bb^ ± 0.00	0.1573 ^Aa^ ± 0.00	0.1007 ^Aa^ ± 0.00	0.0130 ^Cc^ ± 0.00	0.0000	0.1 ^a^ [65]
Heptachlor (mg/kg)	0.0000	0.2175 ^Aa^ ± 0.00	0.2167 ^Aa^ ± 0.00	0.1733 ^Bb^ ± 0.00	0.0000	0.0000	0.01 ^c^ [66]
g-chlordane (mg/kg)	0.1049 ^Aa^ ± 0.00	0.1574 ^Aa^ ± 0.00	0.1551 ^Aa^ ± 0.00	0.1920 ^Aa^ ± 0.00	0.1120 ^Aa^ ± 0.00	0.0000	0.1 ^a^ [64]
Lindane (mg/kg)	0.0000	0.0833 ^Aa^ ± 0.00	0.0802 ^Aa^ ± 0.00	0.0680 ^Bb^ ± 0.00	0.0000	0.0000	0.02 ^c^ [63]
DichloroBiphenyl (mg/kg)	0.0001 ^a^ ± 0.00	0.0000	0.0000	0.0000	0.0000	0.0000	0.2 ^b^ [57]

Key: Each value from triplicate determinations, presented in mean ± standard error (SE); N/A = Not available; values with different superscripts (lowercase [^a–f^]) are statistically significant (*p* < 0.05) compared to the maximum permissible limits; values with different superscripts (uppercase [^A–C^]) are statistically significant (*p* < 0.05) across samples (of same pesticide residue).

**Table 6:** Concentrations of pesticide residues detected in the studied leafy vegetables of *Telfairia occidentalis* leaf, *Amaranthus* leaf, waterleaf, and scent leaf.

Detected Pesticides	*Telfairia occidentalis* Leaf	*Amaranthus* Leaf	Waterleaf	Scent Leaf	Maximum Permissible Limit and Reference Sources
Isopropylamine (mg/kg)	0.0183 ^Aa^ ± 0.00	0.0000	0.0237 ^Ba^ ± 0.00	0.0000	0.1 ^b^ [56]
DichloroBiphenyl (mg/kg)	0.0115 ^Ab^ ± 0.00	0.0062 ^Bc^ ± 0.00	0.0059 ^Bc^ ± 0.00	0.0112 ^Ab^ ± 0.00	0.2 ^a^ [57]
HCB (mg/kg)	0.1233 ^Ba^ ± 0.00	0.0000	0.0000	0.2630 ^Ab^ ± 0.00	0.5 ^c^ [58]
Endosulfan (mg/kg)	0.0548 ^Ab^ ± 0.00	0.0376 ^Bb^ ± 0.00	0.0000	0.0000	0.5 ^a^ [59]
Aldrin (mg/kg)	0.1694 ^Ba^ ± 0.00	0.0000	0.7710 ^Ac^ ± 0.00	0.0000	0.2 ^b^ [60]
Profenofos (mg/kg)	0.6734 ^Ac^ ± 0.00	0.0000	0.0000	0.4860 ^Ba^ ± 0.00	0.5 ^b^ [61]
Carbofuran (mg/kg)	0.0000 ± 0.00	0.0000	0.0024 ±0.00	0.0000	N/A
DDT (mg/kg)	0.2208 ^Bc^ ± 0.00	0.0000	0.0000	0.0718 ^Ab^ ± 0.00	0.01 ^a^ [62]
Lindane (mg/kg)	0.0000	0.5036 ^a^ ± 0.00	0.0000	0.0000	0.02 ^b^ [63]
g-chlordane (mg/kg)	0.0000	0.0642 ^Ba^ ± 0.00	0.1031 ^Ab^ ± 0.00	0.0000	0.1 ^b^ [64]
Dichlorvos (DDVP) (mg/kg)	0.0286 ^Ab^ ± 0.00	0.0199 ^Ab^ ± 0.00	0.0000	0.0003 ^Ba^ ± 0.00	0.1 ^c^ [65]
Heptachlor (mg/kg)	0.0000	0.5023 ^Bc^ ± 0.00	0.0000	0.1258 ^Ab^ ± 0.00	0.01 ^a^ [66]
t-nonachlor (mg/kg)	0.0000	0.1468 ^b^ ± 0.00	0.0000	0.0000	0.04 ^a^ [67]
Chlorpyrifos (mg/kg)	0.0000	0.0000	0.0000 ^a^ ± 0.15	0.0000	1.0 ^b^ [65]

Key: Each value from triplicate determinations, presented in mean ± standard error (SE); N/A = Not available; values with different superscripts (lowercase [^a–c^]) are statistically significant (*p* < 0.05) compared to the maximum permissible limits; values with different superscripts (uppercase [^A,B^]) are statistically significant (*p* < 0.05) across samples (of same pesticide residue).

**Table 7:** Concentrations of pesticide residues detected in the studied fruit vegetables (okro, cucumber, carrot, and watermelon).

Detected Pesticides	Okro	Cucumber	Carrot	Watermelon	Maximum Permissible Limit and Reference Sources
Isopropylamine (mg/kg)	0.0110 ^Aa^ ± 0.00	0.0000	0.0346 ^Ba^ ± 0.05	0.0000	0.1 ^b^ [56]
DichloroBiphenyl (mg/kg)	0.0000	0.0062 ^Aa^ ± 0.00	0.0099 ^Aa^ ± 0.00	0.0000	0.2 ^b^ [57]
HCB (mg/kg)	0.0253 ^Aa^ ± 0.00	0.0000	0.0000	0.0804 ^Ba^ ± 0.00	0.5 ^b^ [58]
Endosulfan (mg/kg)	0.0147 ^Aa^ ± 0.00	0.0376 ^Ba^ ± 0.00	0.0733 ^Ca^ ± 0.00	0.0000	0.5 ^b^ [59]
Aldrin (mg/kg)	0.0000	0.0000	0.2420 ^Ac^ ± 0.00	0.0009 ^Ba^ ± 0.00	0.2 ^b^ [60]
Profenofos (mg/kg)	0.0000	0.0000	0.3304 ^Aa^ ± 0.00	0.2104 ^Ba^ ± 0.00	0.5 ^b^ [61]
DDT (mg/kg)	0.0808 ^Ac^ ± 0.01	0.0000	0.0000	0.0005 ^Ba^ ± 0.00	0.01 ^b^ [62]
Lindane (mg/kg)	0.0000	0.5036 ^Aa^ ± 0.00	0.5171 ^Aa^ ± 0.00	0.4191 ^Aa^ ± 0.00	0.02 ^b^ [63]
g-chlordane (mg/kg)	0.0000	0.0642 ^Aa^ ± 0.00	0.1175 ^Ab^ ± 0.00	0.0000	0.1 ^b^ [64]
Dichlorvos (DDVP) (mg/kg)	0.0000	0.0199 ^Aa^ ± 0.00	0.0166 ^Aa^ ± 0.00	0.0000	0.1 ^b^ [65]
Heptachlor (mg/kg)	0.0232 ^Ab^ ± 0.00	0.5023 ^Cd^ ± 0.00	0.0000	0.2118 ^Bc^ ± 0.00	0.01 ^a^ [66]
t-nonachlor (mg/kg)	0.0000	0.1468 ^Cd^ ± 0.00	0.0003 ^Aa^ ± 0.00	0.0811 ^Bc^ ± 0.00	0.04 ^b^ [67]
Chlorpyrifos (mg/kg)	0.2092 ^Aa^ ± 0.00	0.0000	0.0000	0.3161 ^Aa^ ± 0.01	1.0 ^b^ [65]

Key: Each value from triplicate determinations, presented in mean ± standard error (SE); values with different superscripts (lowercase [^a–d^]) are statistically significant (*p* < 0.05) compared to the maximum permissible limits; values with different superscripts (uppercase [^A–C^]) are statistically significant (*p* < 0.05) across samples (of same pesticide residue).

## Data Availability

Data sharing not applicable.
